# Preparation of Peelable Coating Films with a Metal Organic Framework (UiO-66) and Self-Crosslinkable Polyurethane for the Decomposition of Methyl Paraoxon

**DOI:** 10.3390/polym11081298

**Published:** 2019-08-02

**Authors:** Ngo Hoang Long, Hee-woong Park, Gyeong-seok Chae, Jung Hyun Lee, Se Won Bae, Seunghan Shin

**Affiliations:** 1 Green Chemistry & Materials Group, Korea Institute of Industrial Technology (KITECH), Cheonan, Chungnam 31056, Korea; 2 Department of Green Process and System Engineering, Korea University of Science & Technology (UST), Cheonan, Chungnam 31056, Korea; 3 Department of Chemical and Biological Engineering, Korea University, Seoul 02841, Korea

**Keywords:** metal–organic framework, self-crosslinkable waterborne PU, peelable coating film, methyl paraoxon, decomposition

## Abstract

For the fabrication of a peelable coating material that decomposes methyl paraoxone (MPO), a nerve agent simulant, self-crosslinkable waterborne polyurethanes (PUs) containing silane groups at the ends and a metal organic framework (UiO-66) were synthesized. UiO-66 dispersed PU solutions for spray coating were prepared by controlling the amount of silane in PU and the content of UiO-66. PUs with a large amount of silane (more than 7.2 wt.%) were easily gelated by adding UiO-66 because the solution was changed from neutral (pH = 7.3) to strongly acidic (pH = 2.5). Therefore, the silane content in PUs should be carefully controlled for the fabrication of composite films. When UiO-66 was added to the PU with a silane content of 2.7 wt.%, the reinforcing effect by UiO-66 was observed up to 15.3 wt.%, but a further increase in UiO-66 content decreased both the tensile strength and the elongation. The peel strength of the PU composite films on polyethylene (PET) and glass substrates decreased with increasing UiO-66 content, but their MPO conversion increased with increasing UiO-66 content. The PU composite film with 49.5 wt.% of added UiO-66 showed the MPO conversion of 63.2% and was easily peeled off from PET and glass substrates.

## 1. Introduction

The UiO-66, a type of metal organic framework (MOF), is known to have an excellent activity for the decomposition of compounds with organophosphorus structures [1,2,3,4,5,6,7,8,9]. In particular, high-throughput screening results for solid catalysts for the decomposition of nerve agents, such as methyl paraoxon (MPO), showed that at pH 8, UiO-66 has a more active structure than titania, zeolite, and other polyoxometrics [10,11]. However, UiO-66 is restricted in use due to its particular shape, necessitating the fabrication of composite films with organic binders or nanofibers by electrospinning methods [6,9,12,13,14].

To fabricate composite films with UiO-66, Pinto et al. synthesized UiO-66 directly onto polyurethane (PU) instead of mixing PU and UiO-66 particles [12]. Semino et al. studied the compatibility of the MOF and polymers and reported that polymer rigidity is an important factor for MOF distribution. These authors also demonstrated that polymers with Young’s modulus of less than 1 GPa are preferred for better MOF dispersion [13]. Recently, Kalaj et al. fabricated composite films using UiO-66-NH_2_ and PA-66 but observed a significant decrease in the activity rate for UiO-66-NH_2_ due to compositing with PA-66 [14].

There are several examples of the UiO-66 nanofibers fabricated by the electrospinning method [6,9]. In 2016, Peterson et al. fabricated nanofibers with a UiO-66 solution by electrospinning methods [6]. McCarthy et al. reported that PMMA/Ti(OH)_4_/UiO-66 composite nanofibers prepared by the electrospinning method decomposed 90% of MPO within 2 h at room temperature because TiO_2_ accelerated the hydrolysis reaction by UiO-66 [9]. However, UiO-66-containing nanofibers have limited applications due to their unique shape. UiO-66-containing nanofibers are easily used for fiber mat-based applications, but they are not good for coating applications. Therefore, for use as coating materials, UiO-66 must be distributed over the polymer binder.

When UiO-66-containing polymer coating films are used for decomposing MPO, in some cases, it is necessary to peel off the coating films after decomposition. Unlike chemical agent resistance coating (CARC), which repels chemicals by preventing adsorption, this coating layer decomposes chemicals by UiO-66. The decomposition mechanism of MPO by UiO-66 will be similar to that of dimethyl methylphosphonate (DMMP) proposed by Plonka et al. [15]. They reported that DMMP, which is bound to an undercoordinated Zr atom of the MOF adjacent to a Zr–OH group, decomposes to methyl methylphosphonate by nucleophilic addition of the hydroxide ligand. Therefore, it is needed to peel off the coating layer to prevent secondary contamination by decomposed and adsorbed chemicals, which remain in the coating layer. This peeling off requires the organic binder to be peelable which in turn requires it to have suitable tensile and peel strength values and to exhibit easy removal from substrates. Various liquid solutions and dispersions have been used as peelable coating materials, with waterborne vinyl-acrylic and acrylic copolymer emulsions, poly(vinyl alcohol), and waterborne PU dispersions as representative examples [16,17,18,19,20]. In particular, it has been reported that self-crosslinkable waterborne PU with silane groups at the chain ends resulted in a highly strong, tough, and chemically stable film through the crosslinking reaction during the drying process [21,22,23].

In this research, we fabricated UiO-66-containing peelable coating films using self-crosslinkable waterborne PU as a binder. Based on our previous results that showed that waterborne PUs synthesized with dimethylol propionic acid (DMPA) monomer and poly(tetramethylene ether) glycol (PTMG, Mw = 2000 g/mol) had comparably high MPO decomposition values [24], these compounds were used as an ionomer and a polyol, respectively, for self-crosslinkable waterborne PU synthesis. The peelable coating films were prepared using a spray coating apparatus, and their mechanical strengths, MPO decomposition efficiencies, and peel strengths were measured as a function of UiO-66 content. The changes in the mechanical properties after MPO decomposition were also studied here.

## 2. Experiments

### 2.1. Materials

PTMG (Mw = 2000 g/mol), dimethylol propionic acid (DMPA 98%), isophorone diisocyanate (IPDI, 98%), ethylene glycol (EG, 99.8%), triethylamine (TEA, 99%), and (3-aminopropyl)triethoxysilane (APTES, 99%) were used for PU synthesis. Acetone, 1-Methyl-2-pyrolidinone (NMP), and distilled water were used as solvents. Zirconium (IV) chloride (ZrCl_4_, ≥99.5%), terephthalic acid (TPA, 98%), aqueous hydrochloric acid (HCl, 37%), N,N′-dimethylformamide (DMF), and ethanol were used for the synthesis of UiO-66. MPO, methanol (anhydrous, 99.8%) and N-ethylmorpholine (N-EM, ≥97%) were used for the decontamination experiments. Methyl ethyl ketone (MEK) was used as the solvent for UiO-66 predispersed solution. All of the reagents were purchased from Sigma Aldrich (St. Louis, MO, USA) and were used without further purification.

### 2.2. Synthesis of UiO-66

ZrCl_4_ (200.00 g, 0.86 mol) and TPA (142.48 g, 0.86 mol) were dissolved in DMF (6 L) in a 10 L bottle and stirred using a magnetic bar to achieve a clear solution. After a clear solution was obtained, HCl (94.94 mL, 0.86 mol) was added into the solution. The final solutions were incubated in a conventional oven at 120 °C for 24 h and white precipitates were observed in the bottom of the bottle. The white solids were isolated by centrifugation and washed with DMF (3 times after every 6 h) and ethanol (3 times after every 6 h). Then, the final product was collected by centrifugation and dried in an oven at 80 °C for 12 h. Finally, approximately 200 g of white product was obtained with a yield of approximately 58%.

The obtained UiO-66 showed the average particle size of 190 nm (measured using a particle size analyzer, ELS-Z2plus, Otsuka Electronics, Japan) and surface area of 1466 m^2^ g^−1^ with a pore size of 1.82 nm (derived from the N2 isotherm). The particle size distribution and N_2_ isotherm of UiO-66 are given in Figure S1 in the Supplementary Information (SI). Powder X-ray diffraction (PXRD) pattern of UiO-66 was measured by HP powder XRD (D8 Advance, Bruker, MA, USA) and is displayed in Figure S2.

### 2.3. Synthesis of Dimethylol Propionic Acid Based Silane Terminated Polyurethane Dispersions (DSPDs)

PTMG (224.32 g, 0.112 mol), DMPA (12 g, 0.089 mol) and NMP (40 mL) were introduced into a double jacketed glass reactor equipped with a mechanical stirrer and maintained at 75 °C with argon purge. After 15 min stirring at 300 rpm, IPDI (66.76 g, 0.3 mol) was added dropwise for 20 min, and the reaction mixture was stirred for 3 h. EG (4.96 g, 0.08 mol) was added, and the mixture was stirred for another 2 h. Reaction temperature was decreased to 40 °C, and acetone (200 mL) was added. TEA (9.05 g, 0.089 mol) was added dropwise, the reaction mixture was stirred for 1 h, and then, APTES (8.84 g, 0.04 mol) was added. After 1 h stirring, the reaction mixture was cooled and stirred at 1000 rpm for another 1 h after adding distilled water (588.49 mL). After the reaction, acetone was removed using a rotary evaporator. The solid content of DSPD was adjusted to 35 wt.%. To synthesize the PUs containing different amounts of silane, EG, and APTES were added as shown in Table 1.

### 2.4. Preparation of UiO-66/DSPD Composite Films

UiO-66 particles were pre-dispersed in the MEK solvent using a paste mixer (ARV-310, Thinky, Japan) at 2000 rpm for 10 min. A DSPD solution was added to this mixture and mixed at 2000 rpm for 3 min. The UiO-66 content in mixture solution was varied to 10, 20, 40 and 60 wt.% by changing the concentration of the UiO-66 pre-dispersed solution.

The mixture solution of UiO-66 and DSPD was loaded in a 1.2 mm diameter spray gun (LPH-80, ANEST IWATA, Japan) and uniformly sprayed on a PET film or glass substrate to achieve the average thickness of 150 ± 20 μm. The film was dried at room temperature for 6 h and then dried in an oven at 50 °C for 12 h.

### 2.5. Characterizations

The particle size of DSPD was measured using a particle analyzer (ELS-Z2plus, Otsuka Electronics, Osaka, Japan), and the measurement was repeated 3 times at 25 °C.

The gel fractions of DSPD and UiO-66/DSPD films were determined by Soxhlet extraction using THF as the solvent. Soxhlet extraction was performed for 48 h, and the sample was dried in a vacuum oven at 80 °C until its weight no longer changed. The gel fraction was calculated by comparing the weights of the samples before and after the extraction.

The tensile properties of the DSPD and UiO-66/DSPD films were measured using a Zwick Z005 universal testing machine (Zwick Roell Utm, Germany) at room temperature. Dumbbell-shaped test specimens (60 × 3 × 0.25 mm^3^, length × width × thickness) were used, and the deformation rate was 50 mm·min^−1^. The cross-section morphologies of UiO-66/DSPD composites after tensile tests were observed by FE-SEM (JSM 6701F, JEOL, Japan).

The 180° peel strengths of coated films were measured using a peel tester (SurTa Extra 2A system, Chemilab, Korea). The samples (25 × 100 mm^2^, width × length) were coated on the PET/glass substrates and tested with a rate of 300 mm min^−1^ at room temperature until the peeling length was 70 mm.

The nerve agent simulant MPO was used for evaluating the decontamination properties of DSPD and UiO-66/DSPD composite films. The UV-Vis absorbance of p-nitrophenolate (*λ*_max_ = 407 nm), which is the hydrolysis product of MPO, was used to calculate the decontamination efficiency. A film specimen with a rectangular shape (1 × 1 × 0.015 cm^3^, width × length × thickness) was used for the decontamination test, and other details of the conditions were the same as in previous research [3].

## 3. Results and Discussion

### 3.1. Characterization of DSPD Films

**Scheme 1:**
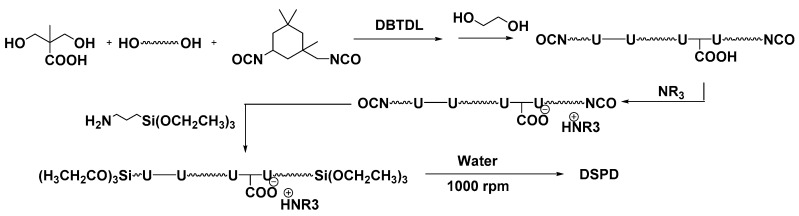
Reaction scheme of DMPA based silane terminated polyurethane dispersion (DSPD).

Silane-terminated PU dispersions were synthesized as shown in Scheme 1. The molecular weight of the reaction products was measured as 3800 g/mol prior to EG addition. The molecular weight was increased to 8800, 6900, 5200, and 4700 g/mol by the addition of EG depending on the silane to EG ratio. As the silane to EG ratio increased, the molecular weights of the PU prepolymer decreased due to the excess isocyanate groups present prior to the silane capping reaction.

**Figure 1:**
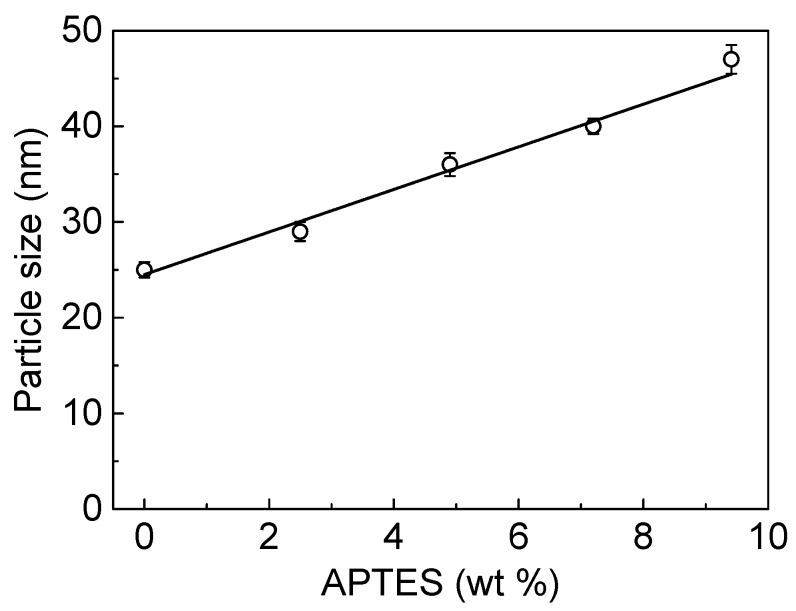
Particle sizes of DSPDs with different amounts of APTES.

The particle sizes of DSPDs varied from 25 to 47 nm depending on the APTES content (see Figure 1). This result is in good agreement with the results reported by Sardon et al. [23]. These authors reported that a small amount of amine silane does not affect the size of the dispersed particles until it exceeds 14 wt.%. For more than 14% amine silane content, an inorganic-rich domain begins to appear on the particle surface, increasing particle size. Considering the total content of amine silane in our system (which is less than 10 wt.% of solid content), the particle size increase by silane addition should be interpreted as being due to urea linkage formation. This phenomenon is because urea linkages that are formed by the reaction of isocyanate and amine silane are less hydrophilic than the urethane linkage.

**Figure 2:**
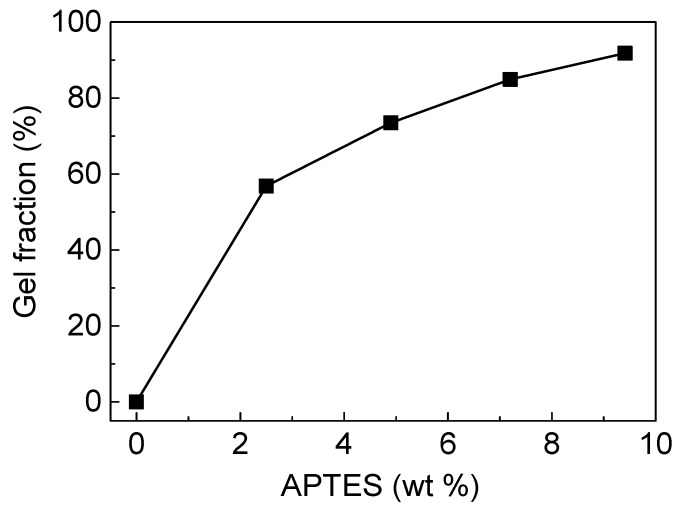
Gel fractions of polyurethane films prepared by DSPDs with different amounts of APTES. Films were dried at room temperature for 48 h and extracted with tetrahydrofuran for 48 h using a Soxhlet extractor.

Self-crosslinking of DSPD was confirmed by measuring the gel content of the cured film. As the APTES content increased, the gel contents of the DSPD films increased from 0 to 91.8% (Figure 2).

**Figure 3:**
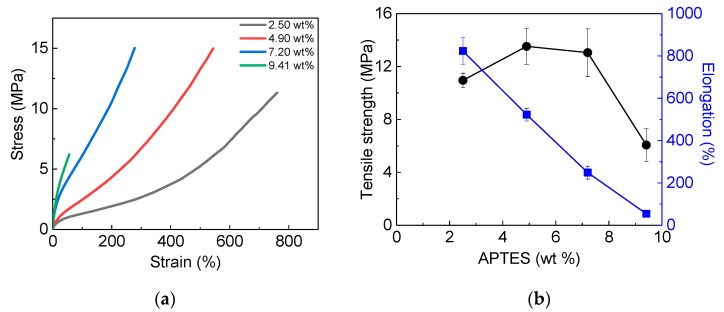
(**a**) Stress–strain curves and (**b**) tensile strengths (●) and elongations (▪) of DSPD films with different silane contents. (DSPD having 0 wt.% of silane could not produce a standalone film due to its low *T_g_*.).

Figure 3a,b show stress–strain curves and tensile strengths and elongations of DSPD films with different amounts of APTES. The elongation at break decreased monotonically with APTES content while tensile strengths increased until 7.20 wt.% of APTES and decreased at 9.41 wt.% of APTES (Figure 3b). Although the film with the APTES content of 9.41 wt.% had the highest gel fraction value, it showed low tensile strength with small elongation. For 9.41 wt.% APTES containing films, the polymer chains between the siloxane bonds are easily disentangled with low strain due to their short chain lengths and begin to endure external stress even at small deformation. Polymer chains are mainly composed of soft PTMG and IPDI, and therefore, they were easily broken at low stress levels.

### 3.2. Characterization of UiO-66/DSPD Composite Films

When UiO-66 particles were dispersed in a DSPD solution, only DSPD containing 2.5 and 4.9 wt.% of APTES did not induce an abrupt viscosity increase and gelation. As the APTES content in DSPD increased, gelation was observed after the addition of UiO-66 within a very short time. This result is because the condensation between the silanol groups of DSPD became fast due to the pH change induced by the UiO-66 particles. The pH of distilled water changed from 7.3 to 2.5 due to the addition of the UiO-66 particles (5 wt.% solution). This value is the similar to the value reported by Bakuru et al. [25].

Therefore, UiO-66/DSPD composite films were prepared with DSPD with 2.5 wt.% APTES as the matrix binder. The UiO-66 content in the composite films was determined by TGA. The actual amounts of UiO-66 in the composite films were 6.5, 15.3, 32.4, and 49.5 wt.% for the films prepared with 10, 20, 40, and 60 wt.%, respectively, of the UiO-66 dispersed DSPD solutions. Based on the TGA analysis, 76.5–82.5% of input amount of UiO-66 were present in the final films after spray coating.

**Figure 4:**
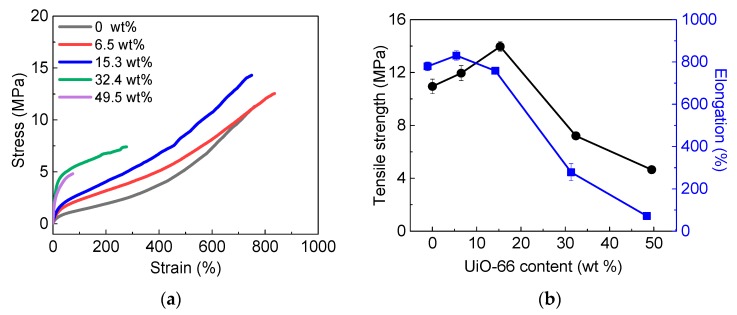
(**a**) Stress–strain curves and (**b**) tensile strengths (●) and elongations (▪) (**b**) of the composite films prepared using DSPDs with different amounts of UiO-66 particles; DSPD has the APTES content of 2.5 wt.%.

Figure 4 displays the tensile properties of the UiO-66/DSPD composite films. The tensile strength of these composite films increased slightly until the UiO-66 content was 15.3 wt.% but decreased remarkably with a further amount of UiO-66.

**Figure 5:**
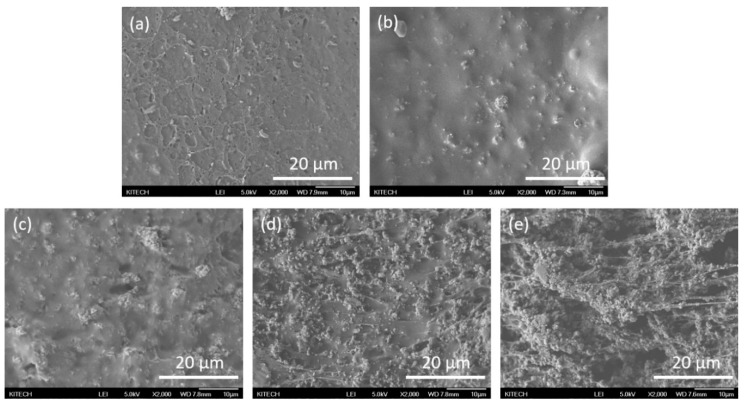
SEM images of the fractured surfaces of the UiO-66/DSPD composite films with (**a**) 0, (**b**) 6.5, (**c**) 15.3, (**d**) 32.4, and (**e**) 49.5 wt.% UiO-66 contents. Magnification is 2000.

The fractured surfaces of the UiO-66/DSPD composite films after the tensile test showed coarse surface and voids that are increased with the increasing amount of UiO-66 (Figure 5). When 32.4 and 49.5 wt.% of UiO-66 were included, the obtained composite films exhibited UiO-66 aggregates and many voids. An examination of the fracture surfaces and tensile strengths of the 6.5 and 15.3 wt.% UiO-66 dispersed composite films shows that tensile strength is improved by the reinforcing effect of the UiO-66 particles at low content level. However, as the amount of UiO-66 increased, DSPD content decreased and became insufficient for creating a fully connected polymer network. In addition, UiO-66 aggregates have a detrimental effect on the mechanical properties because they act as stress concentrators and are weak points for tensile stress.

**Figure 6:**
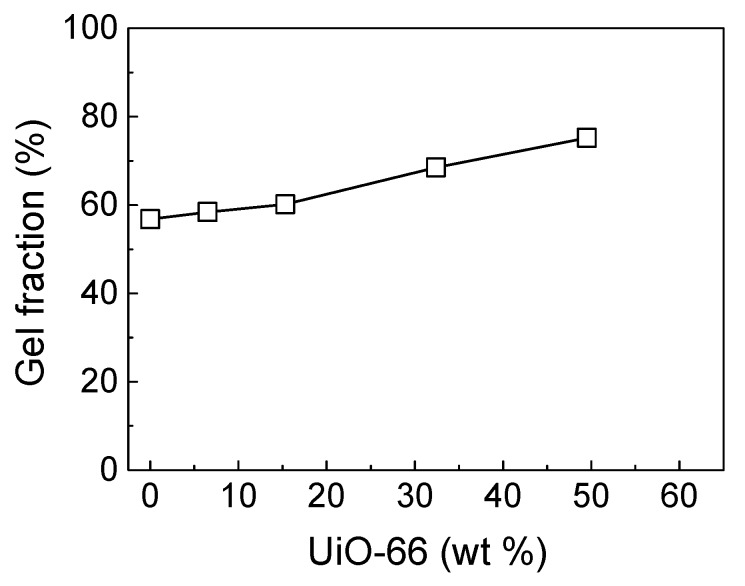
Gel fractions of the UiO-66 dispersed DSPD films as a function of UiO-66 content. (DSPD with 2.5 wt.% silane content was used for composite film fabrication).

Figure 6 shows the gel fractions of the composite films with different UiO-66 contents. Gel fraction increased slightly with increasing UiO-66 content. This increase is also because the siloxane formation reaction was promoted due to the change in pH with increasing UiO-66 content.

### 3.3. MPO Decomposition of Composite Films

**Figure 7:**
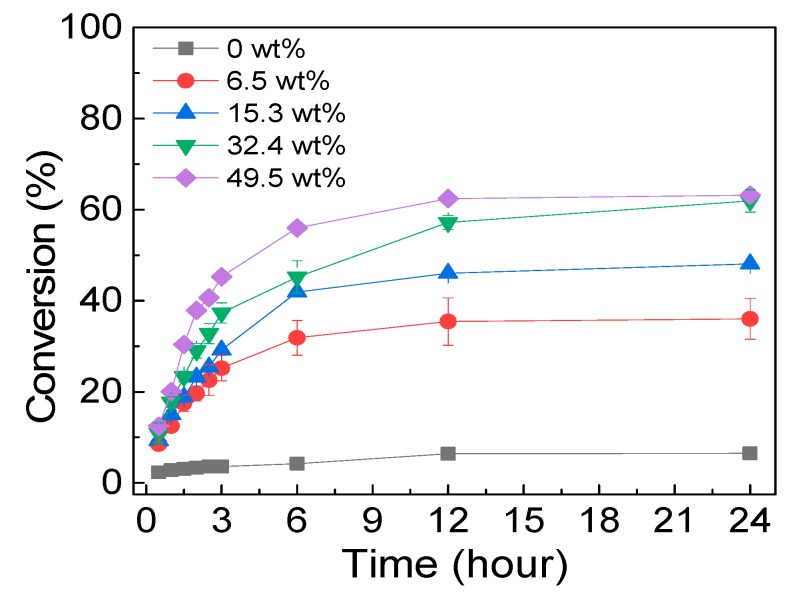
Methyl paraoxone (MPO) decomposition values of UiO-66/DSPD composite films with different UiO-66 contents.

The MPO decompositions by the composite films were determined using the method of Katz et al. [3]. UV-Vis spectroscopy was used for monitoring the nitrophenolate ions. As the reaction time increased, the peak at 407 nm indicating nitrophenolate formation increased (see Figure S3). The conversion profile for the MPO to nitrophenolate was calculated using the absorbance of the nitrophenolate peak. Figure 7 shows that MPO decomposition increased with increasing UiO-66 content and reaction time. After 6 h reaction, the MPO conversion rates of all composite films decreased, and MPO conversion at 24 h was estimated to be approximately 36.0–63.2% depending on the UiO-66 content. The decrease in the MPO conversion rate with reaction time is due to the activity degradation of UiO-66 by the high pH of the test solution (pH = 12). It is well-known that UiO-66 loses its activity in basic solutions [26,27]. In this study, UiO-66 particles were degraded by co-catalyst (N-EM) (see Figure S2). However, UiO-66 particles dispersed in a polymer film were less influenced by N-EM than UiO-66 slurry considering our previous results [24]. Based on the SEM images showing the porous surface and voids of the 49.5 wt.% UiO-66 dispersed film (Figure 5), the test solution can easily diffuse into the film and is decomposed rapidly at the early reaction time, but it gradually degrades the activity of UiO-66.

The rate constant of MPO decomposition was calculated using pseudo-first order reaction kinetics (Figure S4). The pseudo-first-order rate constant, *k*, is also increased by the amount of UiO-66 in the composite films.

### 3.4. Peeling Properties of UiO-66/DSPD Composite Films

**Figure 8:**
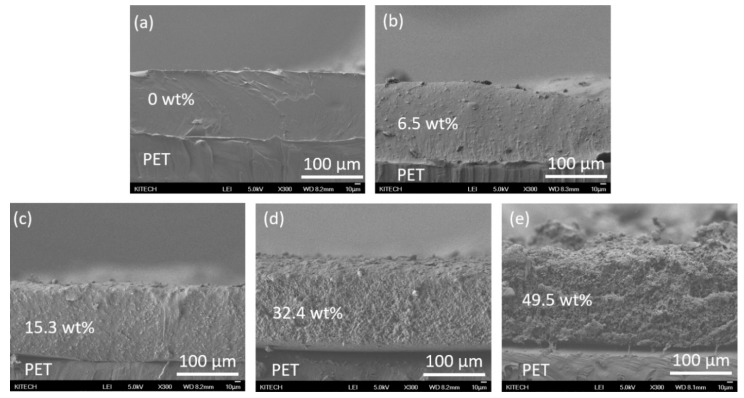
Cross-section SEM images of the UiO-66/DSPD composite films with (**a**) 0, (**b**) 6.5, (**c**) 15.3, (**d**) 32.4, and (**e**) 49.5 wt.% UiO-66 contents on polyethylene (PET) substrates.

The peeling properties of the DSPD and UiO-66/DSPD films were measured by the 180° peel test method. DSPD and UiO-66/DSPD films were prepared on glass and PET substrates by the spray-coating method. The average thickness of the coated films was 150 ± 20 μm. As shown in Figure 8, the film thickness deviation increased with UiO-66 content.

**Figure 9:**
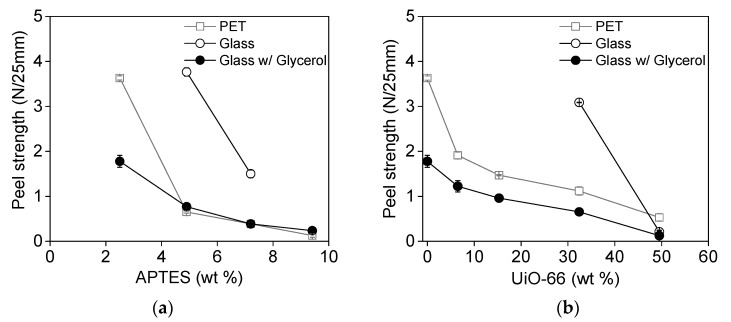
(**a**) Peel strengths of DSPD films with different amounts of silane and (**b**) DSPD composite films with different amounts of UiO-66 (**b**) on the PET or glass substrate. The silane content of composite films is 2.5 wt.%.

Figure 9a shows the peel strengths of DSPD on a PET or glass substrate as a function of silane content. The remarkable decrease in the peel strength with APTES content is related to film elongation. As more silane is included, DSPD films become harder, and the applied stress was used for peeling rather than for elongating the film. Compared with a PET substrate, DSPD showed a higher peel strength on a glass substrate. DSPD with the APTES content of 2.5 wt.% was not peeled off from the glass substrate due to the strong interaction with the glass surface. As the APTES content increased further, peel strength decreased and was not obtained at 9.41 wt.% of APTES due to film brittleness. To reduce the peel strength on the glass substrate, glycerol was added as a releasing agent. According to the study by Lewandowski et al., glycerol is a good agent that reduces the peel strength of self-crosslinkable PU by migrating to the coating-substrate interface [22]. Glycerol was mixed well with the DSPD solution to provide a clear and defect-free coating. After the addition of 5 wt.% of glycerol, the peel strength of the DSPD film with 4.9 wt.% of APTES on the glass substrate decreased from 3.77 to 0.77 N/25 mm. Therefore, aqueous or non-aqueous stripping or washing solution was not required to remove the coating film from the glass substrate.

The peel strengths of DSPD films were further decreased with the addition of UiO-66 (Figure 9b). The DSPD film containing 2.5 wt.% of silane on the glass substrate was peeled off by the addition of 32.4 wt.% of UiO-66. This observation is also because of the increased modulus obtained by compositing with UiO-66. Therefore, the film was easily peeled off with a relatively small stress.

### 3.5. Mechanical Property Changes after MPO Decomposition

The tensile strengths of the UiO-66/DSPD composite films were measured after the MPO decomposition test. The middle parts of the composite films were soaked in the test solution as shown in Figure S5. After a certain time, the composite films were removed from the solution and dried at room temperature for 3 h prior to the tensile test.

**Figure 10:**
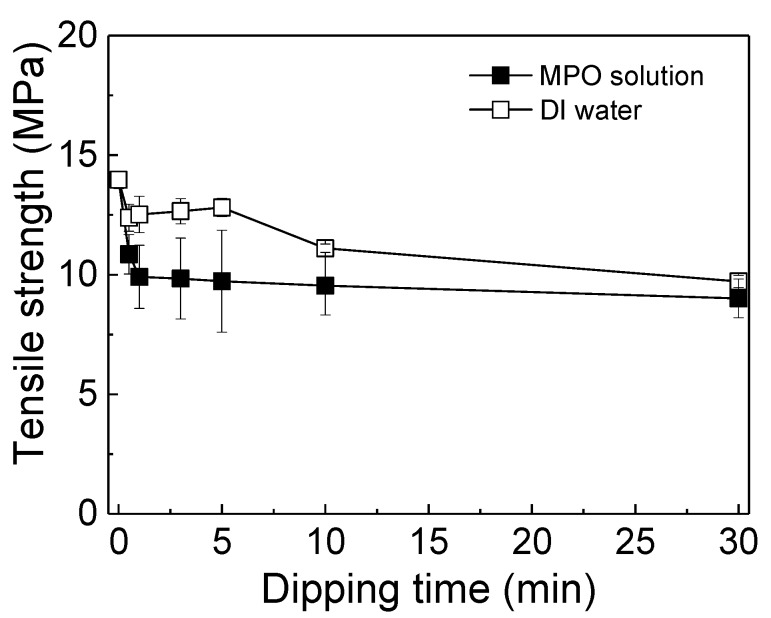
Tensile strengths of the UiO-66/DSPD composite films as a function of dipping time. The composite film had the UiO-66 content of 15.3 wt.%, and tensile tests were performed 3 h after dipping.

Figure 10 shows that the tensile strength of a composite film decreased with dipping time. Tensile strength decreased remarkably after 1 min dipping but was only marginally changed after 5 min. In our experimental conditions, the UiO-66/DSPD composite film maintained 71% of its original tensile strength after 5 min dipping. The decrease in the tensile strength is attributed to the water and reactants in the MPO solution that are diffused into the composite films acting as plasticizers.

Considering that DSPD showed better polar-solvent resistance than the waterborne PU dispersions without silane groups due to its self-crosslinking property (see Figure S6), the tensile strength of the UiO-66/DSPD composite films is expected to be maintained to some extent due to the crosslinked structure.

## 4. Conclusions

This study sought to develop a peelable coating material that can decompose MPO, a nerve agent simulant, using UiO-66 and the self-crosslinking PU binder. Water-dispersible PU materials with silane terminal groups were synthesized as a self-crosslinking PU binder. UiO-66/PU composite films were prepared with PU containing 2.5 and 4.9 wt.% of silane because the siloxane reaction was accelerated by the addition of UiO-66. PU with the silane content of 2.5 wt.% was an appropriate binder for the high loading of UiO-66.

UiO-66/PU (with 2.5 wt.% of silane) composite films prepared by spray coating showed high MPO conversion (63.2% at 49.5 wt.% of UiO-66) but decreased tensile strength and elongation. The composite films also showed low peel strength on the glass and PET substrates. Although it was difficult to peel off the composite films with a low amount of UiO-66 from the glass substrate, this drawback was overcome by the addition of glycerol. The mechanical properties of UiO-66/PU composite films decreased after the MPO decomposition test. After 5 min dipping, 71% of the original value was retained. This rather encouraging result was due to the crosslinked structure of PU.

The present research is the first study to report a peelable coating material with MPO decomposition properties. Therefore, further research is required to examine the long-term stability of UiO-66/PU mixture solutions and to modify the polymeric binder to obtain higher MPO conversion without N-EM.

## Figures and Tables

**Table 1:** Basic compositions of dimethylol propionic acid (DMPA) based silane terminated polyurethane dispersions (DSPDs).

Sample	Polyol (g)	DMPA (g)	IPDI (g)	EG (g)	APTES (g)	EG/APTES (mol ratio)	APTES in Feed (wt.%)	APTES by TGA (wt.%)
DSPD1	224.32	12.00	66.76	4.96	8.84	2	2.79	2.50
DSPD2				3.72	17.68	0.75	5.45	4.90
DSPD3				2.48	26.52	0.33	7.99	7.20
DSPD4				1.24	35.36	0.125	10.41	9.41

## Supplementary Materials

The following are available online at https://www.mdpi.com/2073-4360/11/8/1298/s1.
