# Supplementary material for: Preparation of Peelable Coating Films with a Metal Organic Framework (UiO-66) and Self-Crosslinkable Polyurethane for the Decomposition of Methyl Paraoxon
Source: Polymers (Basel). 2019 Aug 2;11(8):1298. doi: 10.3390/polym11081298 (PMC6722878; doi:10.3390/polym11081298)
Supplement: Supplementary file 1 [file polymers-11-01298-s001.pdf]

Supplementary Information

Preparation of peelable coating films with a metal organic framework (UiO-66) and self-crosslinkable polyurethane for the decomposition of methyl paraoxon

Ngo Hoang Long[1,2], Hee-woong Park[1,3], Gyeong-seok Chae[1,2], Jung Hyun Lee[3], Se Won Bae[1], Seunghan Shin[1]

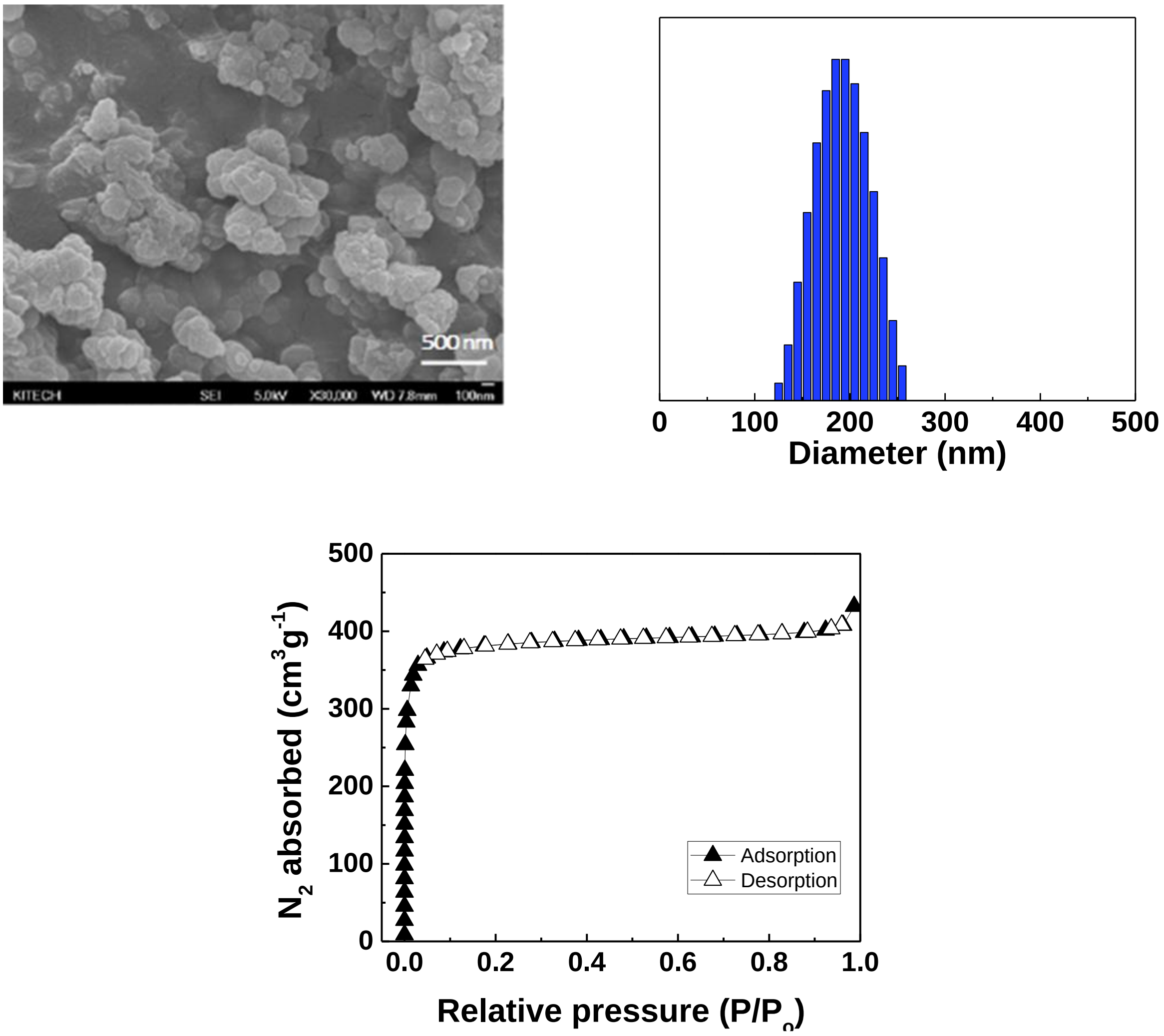

Figure S1. The FE-SEM image, average size and N_2 isotherm of synthesized UiO-66.

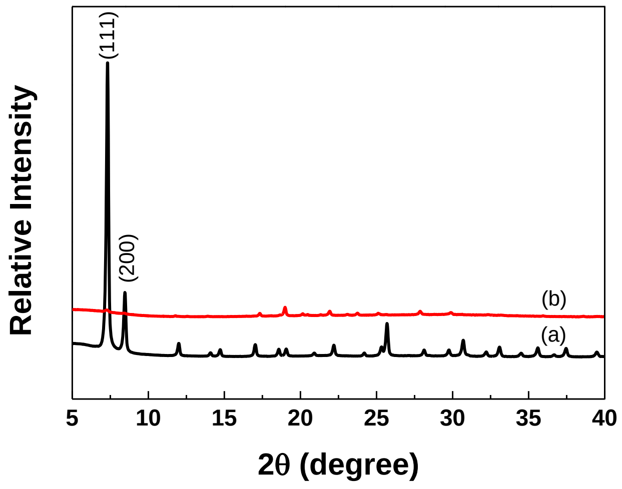

Figure S2. PXRD patterns of UiO-66 particles: (a) pristine and (b) immersed in 0.45 mol/L N-ethyl morpholine aqueous solution for 24 h

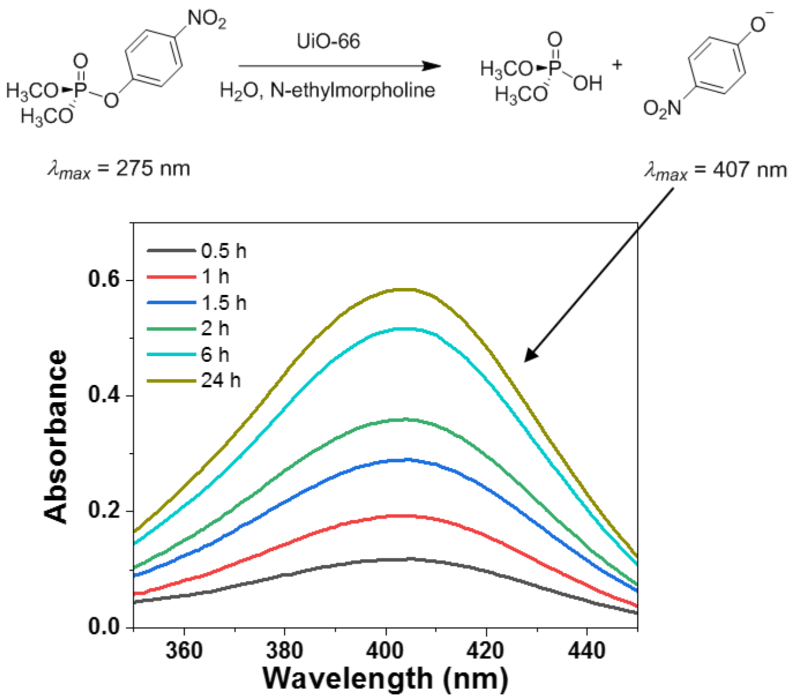

Figure S3. Hydrolysis of methyl paraoxon (MPO) and UV-Vis spectra of the degradation of MPO by a 49.5 wt% UiO-66-dispersed DSPD film as a function of the treatment time.

Experimental method

The rate constant of MPO decomposition was calculated by using pseudo-first order reaction kinetics. The equation $\ln(C_t/C_0) = -kt$ was used, where C_t is the concentration of MPO at time t, C_0 is the initial concentration of MPO, and k pseudo-first-order rate constant. The rate constant of MPO decomposition is increased by the amount of UiO-66 in the composite films.

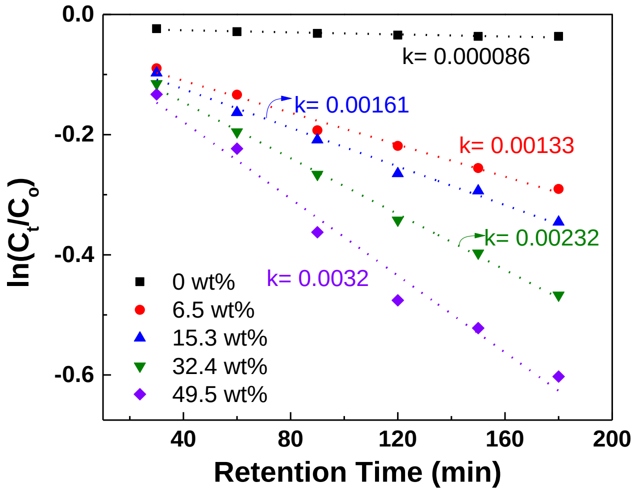

Figure S4. The ln (C_t/C_o) *vs* t plot for MPO decomposition (dot line are calculated by linear fit).

Experimental method

Dumbbell-shaped UiO-66/DSPD composite films were immersed in the test solution for 0.5, 1, 3, 5, 10 and 30 min, respectively, and then they were dried for 3 h prior to tensile test.

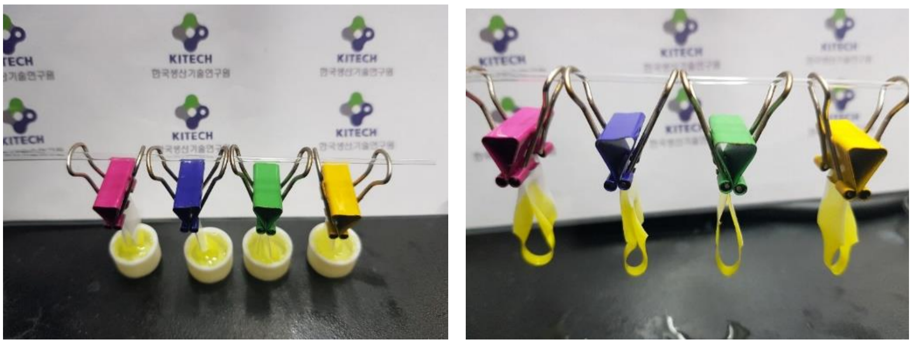

Figure S5. Sample preparation for measuring the mechanical property changes of UiO-66/DSPD composite films after MPO decomposition test.

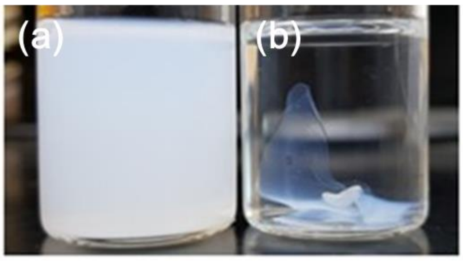

Figure S6. Polar solvent (methanol) resistance of (a) DSPD films without APTES and (b) DSPD films containing 2.5 wt% of APTES.
